# Developing reference criteria for the ecological status of West African rivers

**DOI:** 10.1007/s10661-017-6360-1

**Published:** 2017-12-02

**Authors:** Idrissa Kaboré, O. Moog, A. Ouéda, J. Sendzimir, R. Ouédraogo, W. Guenda, A. H. Melcher

**Affiliations:** 1 Laboratoire de Biologie et Ecologie Animales (LBEA), Université Ouaga I Professeur Joseph Ki-Zerbo, Ouagadougou, Burkina Faso; 2 Centre for Development Research, Institute of Hydrobiology and Aquatic Ecosystem Management, BOKU University of Natural Resources and Life Sciences, Vienna, Austria; 3 International Institute for Applied Systems Analysis (IIASA), Laxenburg, Austria; 4 Ministère de la Recherche Scientifique et de l’Innovation, Institut de l’ Environnement et de Recherches Agricoles (INERA), Ouagadougou, Burkina Faso

**Keywords:** Multiple pressures, Reference conditions, Arid, Rivers

## Abstract

Awareness of sustainable management of water and its biological resources is rising in West Africa, but application of effective tools for biomonitoring and detecting habitats at risk in aquatic ecosystems is limited. In this study, we provide key environmental descriptors to characterize reference sites by applying the following “a priori criteria” (physical and chemical, hydro-morphological, and land use parameters) by exploring their potential to determine suitable reference sites. Using data collected from 44 sites, we identified 37 criteria that reliably identify reference conditions in semi-arid rivers by reflecting the impacts of multiple pressures ranging from low to very high intensity of human uses and impairments. We integrated all these impacts in an overall pressures index, which showed that protected areas can reasonably be considered as credible reference sites as far as they show low overall impact levels from cumulative pressures. We recommend that development of bio-indicator standards should be based on the collection and integration of all the available information, especially quantitative, spatially-explicit data, from benthic macroinvertebrates and fish. Rigorous standardization of bio-indicator protocols will make them more easily applicable for management and conservation of aquatic ecosystem resources in semi-arid zones of Africa.

**Electronic supplementary material:**

The online version of this article (10.1007/s10661-017-6360-1) contains supplementary material, which is available to authorized users.

## Introduction

In Burkina Faso (BF), high water demand due to a high population growth rate and low management capacity has led to overuse of surface water. Two major factors affecting BF river systems are urbanization and agriculture activities (Ouédraogo 2010; Melcher et al. 2012; Kaboré et al. 2015), and several mining activities. Their combination lowers water quality by depositing untreated domestic waste in the rivers and their tributary creeks and channels. In addition, BF river flow regimes have been altered as increasing water demand required dam construction on rivers to establish a water storage network of reservoirs. These alterations to Burkina Faso catchments and their channels have resulted in new water flow and sediment regimes in the rivers and hence a net change in their ecological status. Despite the pressing need to preserve these water resources for human uses and to maintain the biotic integrity of riverine ecosystems, few studies (Guenda 1996; Sanogo et al. 2014) have addressed the ecological status of aquatic ecosystems in Burkina Faso or the means to assess that status, e.g., biomonitoring.

The purpose of biomonitoring in aquatic ecosystems is to evaluate the effect of human activities on biota and the resources they depend on. Several techniques are used in aquatic ecosystem biomonitoring programs, including saprobic techniques (from Kolkwitz and Marsson 1902 to Rolauffs et al. 2004), diversity indices (Metcalfe 1989), biotic indices and scores (Armitage et al. 1983; Dickens and Graham 2002; Ofenboeck et al. 2010; Kaaya et al. 2015) multivariate techniques (Norris and Georges 1993; Kokes et al. 2006), and multimetric indices (Barbour et al. 1995; Hering et al. 2006).

One important component of the biological assessment of stream conditions using macroinvertebrate communities is an evaluation of the direct or indirect effects of human activities or disturbances (Hering et al. 2006; Moog et al. 2008).

The reference condition approach is one of the most effective techniques for biomonitoring and assessing the ecological status of aquatic ecosystems. Thus, every bioassessment approach requires the identification of reference sites and reference conditions (Wright et al. 1984; Resh 1995). According to Barbour et al. (1996), Roux et al. (1999), Ollis et al. (2006), and Stoddard et al. (2006), the reference condition (1) is defined as “the condition that is representative of a group of minimally disturbed sites organized by selected physical, chemical, and biological characteristics” and (2) represents the expected condition for a particular biotic component and acts as a benchmark against which data from a monitoring site is compared. With the reference condition approach, the biological community of a potentially stressed waterbody is compared with that of relatively undisturbed reference sites that have similar environmental conditions. However, several authors pointed out that reference conditions must be systematically identified because all ecosystems experience some level of human disturbance, and truly pristine sites are virtually nonexistent (Thorne and Williams 1997; Wallin et al. 2003). A number of methods can be used to establish the reference condition (Rosgen 1998; Apfelbeck 2001). Some of these methods include extensive spatial survey, predictive modeling, historical data, and expert judgment (Dallas 2000a, b; Alonso et al. 2011). Each method of determining the reference condition has its own strengths and weaknesses (Economou 2002; Sommerhäuser et al. 2003), and each method relies on ecosystem classification to some degree (Wallin et al. 2003; Alonso et al. 2011; Johnson et al. 2013).

In some geographical areas, authors have developed “a priori criteria” to distinguish a reference site from impaired sites, and these criteria were based on different pressures derived from human activities that can affect ecological conditions (Moog and Sharma 2005; Du Preez and Rowntree 2006; Alonso et al. 2011). The criteria selected as “a priori” should define the lowest level of environmental disturbance caused by human activities (Stoddard et al. 2006), and most of these criteria should be fulfilled by selected reference sites to clearly define the reference ecosystem as one that is “acceptably healthy” according to current policy goals (Bailey et al. 2004; Alonso et al. 2011). The criteria for appropriate reference sites may vary among regions, water bodies, and habitat types. However, the most commonly used criteria include physicochemical parameters, hydro-morphological characteristics, land use pattern, and riparian vegetation (Moog and Stubauer 2003; Nijboer et al. 2004). In developing countries where research resources and historical knowledge are limiting factors, only a few studies used abiotic and riparian vegetation criteria (Moog and Sharma 2005; Lakew and Moog 2015) to describe the characteristics of sites. We believe that testing and developing ecosystem health assessment tools are the only ways to rigorously account for the unique characteristics of a novel geographical area. In effect, the lack of robust surface water quality monitoring tools can lead to wasted investment and a failure to implement effective pollution control measures (Lakew and Moog 2015). However, our knowledge of ecological evaluation using comprehensive environmental data on the pressures and the interactions of pressures remains very poor.

The present study establishes new basis of a monitoring program in semi-arid area by setting criteria based on a literature research and adapting the multiple pressure index to identify what low levels of alteration in environmental variables can still support aquatic communities that are relatively intact ecologically.

## Material and methods

### Study area

**Fig. 1:**
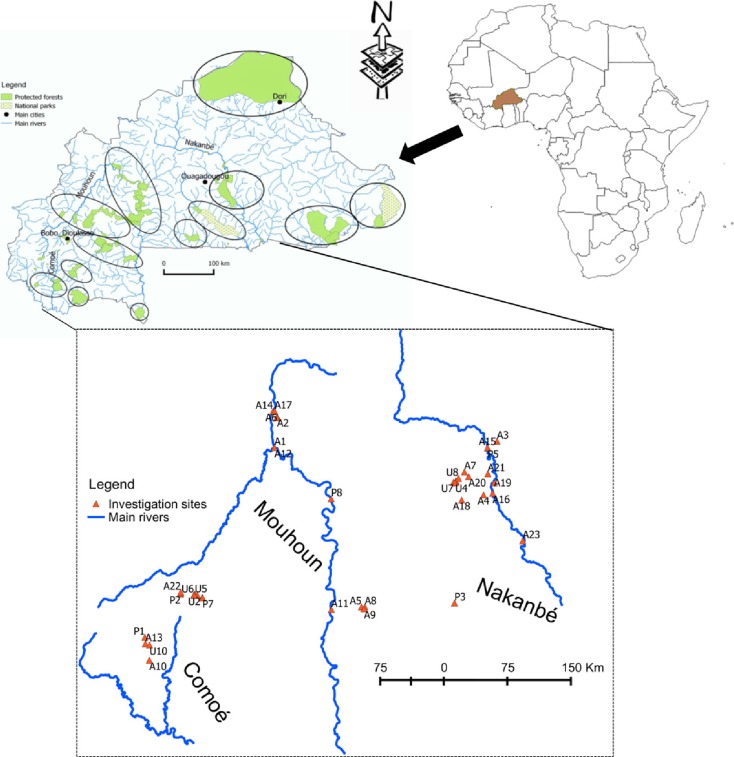
Map of Burkina Faso showing the study area. Circles indicate the protected areas (adapted from BNDT 2009)

Burkina Faso is located in the central part of West Africa (09°20′N & 15°03′ N; 02°20′ E 05°03′ W). The climate is tropical and semi-arid with a temperature range varying between maximum (40 °C) and minimum (16 °C) (Ly et al. 2013; http://www.burkina-faso.climatemps.com/). Three main catchments constitute the hydrological network of Burkina Faso (Fig. 1): Niger, Comoé, and Volta. In Burkina Faso, surface water resources are rain-fed. Two seasons, induced by the northward and southward oscillations of the Intertropical Convergence Zone (ITCZ) front, govern water availability in the country: a relatively short (3–4 months) rainy season with abundant, patchy rainfall during storm events inducing more runoff than infiltration and a relatively long (8–9 months) dry season where no rainfall occurs but temperatures and evaporation are high. To buffer this temporal variability in Burkina Faso, some 2000 reservoirs (MEE 2001) regulate water availability for population and livestock. The total volume of these reservoirs was estimated in 2001 by the GIRE project to be 2.66 billion m^3^ of water at their maximum capacity with an approximate total area of 100,000 ha. The average annual runoff volume (period 1961–1999) of the national river basins is estimated at 7.5 billion m^3^, and the average storage potential of surface water per year is 8.6 billion m^3^ (Sandwidi 2007).

In the early 1970s, severe droughts struck the Sahel and revealed Burkina’s vulnerability to years of low precipitation. Following these droughts, Burkina Faso’s water policies have been primarily oriented toward ensuring a basic supply for all so as to minimize the vulnerability to spells of low precipitation (Ministère de L’Environnement et du Développement Durable [MEDD] 2011). In an ongoing response to the threat of droughts in the 1970s and 1980s, Burkinabe water management institutions continued to proliferate (MEE 2001). Attempts were made to consolidate the various institutions in the 1980s, but real institutional integration started in 1990s (MEE 2011). In 1995, the government of Burkina Faso created the Water and Environment Ministry (MEE). As a result, water-related activities and interventions in the country achieved much greater organization and coordination (MEE 2011). The final stage of reconsideration of the first round of political decisions was reached with the adoption in 1998 of the document on the national policies and strategies of water resources (Sandwidi 2007). Together with this, the GIRE project was established in 1999 to integrate water resources management as recommended in the Dublin and Rio international conferences on water and environment. In the same year (1998), the national water law was put into force, and this new water law recognizes that basic human and environmental needs should be met [Gestion Intégrée des Ressources en Eaux (GIRE) 2001]. Efforts to extend protection to fragile aquatic ecosystems and riverbanks established the Water Law (Assemblée Nationale 2001). It also defined the river catchment area as the geographical unit of water resources management (United Nations Environment Programme-Global Environment Facility [UNEP-GEF] 2012). Despite a proliferation of policies and regulations concerning management of water and associated biological resources, governance has proven inadequate to make fisheries sustainable and is badly in need of the biomonitoring tools that can be used to monitor environmental conditions (Ouédraogo 2010; Sustainable Management of Water and Fish Resources Consortium [SUSFISH] 2015). Such tools are essential to setting the ecological objectives used to formulate and apply policies for sustainable management of fisheries resources. Our study was undertaken in rivers belonging to three catchments: the Nakanbé (former White Volta catchment) in the central part of Burkina Faso (area ca. 70,000 km^2^), the Mouhoun (former Black Volta catchment) in the west (92,000 km^2^), and the Comoé in south-west part of Burkina Faso (18,000 km^2^). The 44 sampling sites selected here fell within two continua ranging from low to very high intensity pressures (Kaboré et al. 2015). Floodplain land use types were defined as follows: “protected” (P), “agricultures”: extensive and intensive agricultures (A), and “urban” (U) including park (UP) of Ouagadougou according to Bondaz (2013) and Kaboré et al. (2015) (Fig. 1).

## Characterization of pressures

**Fig. 2:**
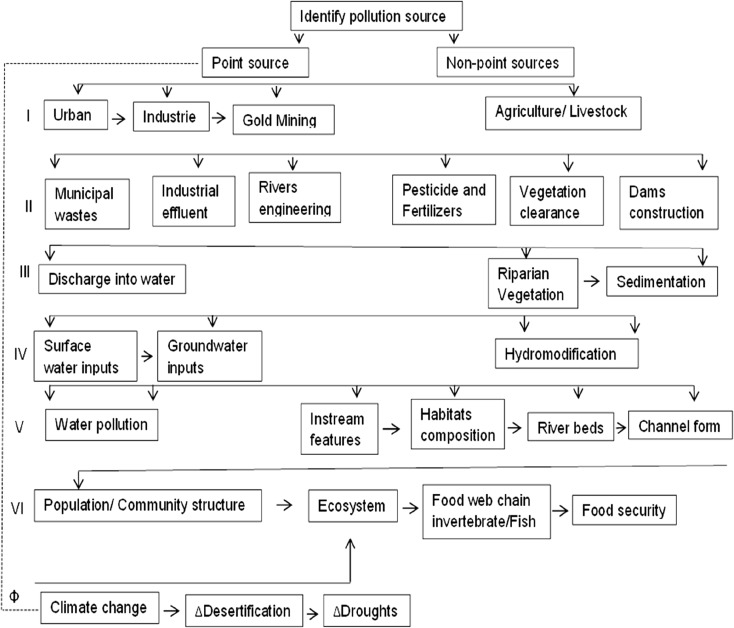
Conceptual diagram illustrates interconnected associations used to visualize the impacts of ecosystem alteration on the biological condition of streams/rivers. (I=drivers; II=pressures, III–V=impacts; VI=reaction, and Ф=natural drivers (adapted from Ziegler et al. 2015)

We characterized pressures and developed an overview of driving forces, pressures, and possible impacts affecting water body quality in Burkina Faso by compiling a list of human disturbances of rivers based on expert opinion and literature reviews (Ouédraogo 2010; MEE 2011; Koblinger and Trauner 2013; link: susfish.boku.ac.at). The interconnected associations used to visualize the impacts of ecosystem alteration on the biological condition of streams and rivers detected in Burkina Faso are shown in conceptual diagram (Fig. 2). This diagram synthesizes evidence of causes and effects in environmental systems where research is conducted to inform policy makers and managers (Ouédraogo 2010; Sendzimir et al. 2015; Kaboré et al. 2015). It offers a basis for objective assessment of available evidence, but also by suggesting potential relations between factors across levels, e.g., driver and impacts.

Setting criteria for some observed pressures may not be too difficult and can be approached from different perspectives. For example, the intensity of point source pollution and the magnitude of its impact can be determined by observing the distribution of the sources in a watershed or by direct measurements of the concentration of pollutants in the water column. Similarly, land use patterns in the riparian zone of study sites can be obtained from local land use maps or remote sensing imagery and geographic information systems (GIS). Nevertheless, developing indicators based on quantifying human pressures and their impact levels remains a challenge. It requires detailed analysis of appropriate data sets that rigorously document local conditions and then can help to establish trends of the current health status of the aquatic ecosystem.

### Definition of reference criteria

#### Hydro-morphological criteria

**Fig. 3:**
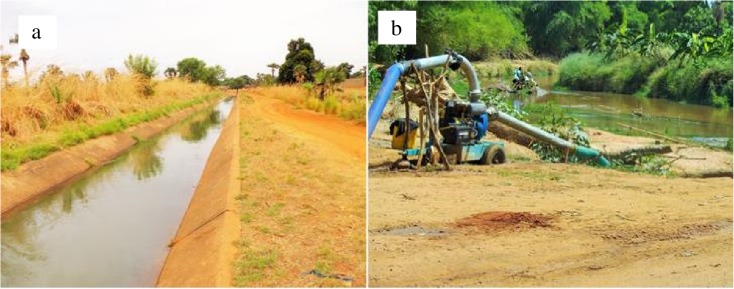
Human pressures on rivers hydro-morphology. **a** Engineering channel and **b** water abstraction by pumping

Developments such as roads, settlements, farm infrastructure, reservoirs, and dams shape our landscape and can impact the ecological functions of water bodies. To characterize and describe those impairments, hydro-morphological tools were used to assess physical aspects of water bodies with a focus on habitat structure and hydraulic features. Hydro-morphological properties of streams reflect interactions between morphology and hydrology that influence the ecological integrity of flowing water ecosystems (Rosgen 1998; Mühlmann 2010). Human modification of natural hydrologic processes disrupts the dynamic equilibrium between the movement of water and the movement of sediment (Poff et al. 1997; Dallas 2000a, b). Indeed, many rivers have been subjected to channelization and artificial levee construction, reducing rivers to single-thread channels and isolating them from their floodplain (Mattingly et al. 1993). In Burkina Faso, major human alterations of hydrology and morphology are caused by damming (e.g., reservoir construction), diversion, water abstraction, and river channelization, respectively (Fig. 3a, b). High water demands during the dry season accelerate drying out of most streams and decrease the discharge of the few perennial rivers. River channelization and the effects of diversion and water abstraction have significant effects on the downstream environment, as well as channel features. Siltation/erosion of rivers caused by removal of riparian vegetation, gravel extraction, and sand excavation constitutes major sources of morphological change.

To address these impacts, beneficial management programs, including river restoration or holistic engineering, are increasingly expected to maintain and restore ecosystem health while also supporting varied human uses (Barrett et al. 2006; Bernhardt and Palmer 2011). Therefore, hydro-morphological parameter groups of the sites defined here could be considered suitable as an ensemble that defines the complete set of hydro-morphological conditions necessary for ecosystem functioning. The said parameter groups can be used to translate into explicit and objective criteria. These criteria address all the relevant structural aspects for the preservation of biotic integrity in stream or river systems (Sánchez-Montoya et al., 2009; Mühlmann 2010). Thus, many studies have found that key hydrology and channel form parameters can be used as solid basis to guide and improve river management strategies and restoration schemes (Bailey et al. 2004; McEnroe et al. 2010; Palmer et al. 2014). The range of pressure criteria agreed with those reported by the authors in Table 1 that embrace all major pressures affecting surface water ecosystem in the study area.

**Table 1:** Variables measured to reflect different pressures on Burkina Faso river systems

Categories	Variables	Characteristic
Morphological pressures	Bed dynamics	Ordinal (5)
	Channel form	
	Bank dynamics	
	In-channel features	
	Channel structure	
Habitat pressures	Substrate composition	
	Riparian vegetation	
Hydrological pressures	Hydrograph and discharge regime	Binary (yes/no)
	Water extraction for hydropower and industrial uses	
	Water extraction for irrigation	
	Dyke for flood altered lateral connectivity between river and riparian zone	
Connectivity pressures	Barrier or reservoir upstream at 100 m of sites	
	Sealing of the river bottom (pavement, concrete)	
Water quality pressures	Point source pollution	Binary (yes/no)
	Artificial eutrophication	
	Known or expected diffusion input	
	Ferro-sulfide reduction	
	Waste dumping into the river or river banks	
	Foam	
	Water foam (except natural sources)	
	Water turbidity (except natural sources)	
	Water odor	
	Fungi and stuffs	
	Conductivity	Linear
	Dissolved oxygen	
	Salinity	
Direct pressures	Cattle washing/watering	Binary (yes/no)
	Livestocks at 100 m of site	
	Sand or gravel excavation	
Riparian land use pressures	Crop farming in the riparian zone	
	Irrigated agriculture	
	Urbanization, industry, and other uses	
	Fishery area	

#### Land use and flooded area-cover criteria

**Fig. 4:**
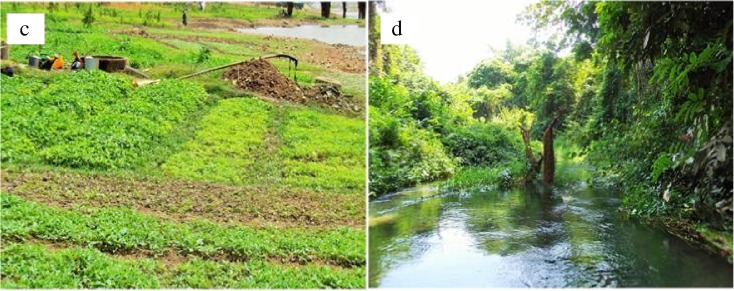
Rivers floodplain use. **a** Crops farming and **b** the protected area

As one of the main drivers of chemical and sediment inputs to surface waters, land use influences water quality. However, Bald et al. (2005) have demonstrated that these influences on water quality could be attributed to the transport capacity of the watershed and the influence of riparian buffers. In Burkina Faso, rivers are impaired practically by a variety of uses that are either aquatic (intense fisheries) or on land, including agriculture, urban, etc. The riparian areas of river basin watersheds are increasingly characterized by intense agricultural usage and human population density (UNEP-GEF 2010). Burkina Faso officially recognizes the problem of rapid population growth (3% in 2013) as a major factor for land use changes and depletion of natural resources (MEDD 2011). Currently major land use changes in the country result from expansion of areas used for crops (cotton, cashew, etc.), livestock, irrigation, and urbanization (Fig. 4c). However, severe negative impacts on benthic macroinvertebrate diversity and drastic change of river morphology are expected due to land use intensification (Cunha et al. 2010; Egler et al. 2012). Riparian vegetation cover is currently restricted to state protected areas, including national parks. “Protected” areas were exposed overall to the lowest levels of human impact, relatively (Kaboré et al. 2015, see Fig. 4d). In a protected area, small bushes, shrubs, and perennial grasses are dominant, but trees are not uncommon. Local riparian vegetation plays a crucial role in nutrient uptake, organic matter, and food supply, as well as in river bank stabilization. Increased lateral connectivity between riparian vegetation and flooded areas enlarges the ecological niche for aquatic animals, and by providing more opportunities for food, nurseries and shelter may constitute a refuge area for a variety of wild terrestrial fauna.

#### Physicochemical criteria

**Fig. 5:**
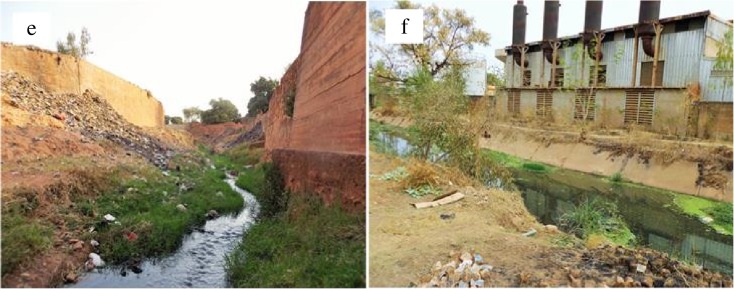
Rivers source pollution. **a** Domestic waste and **b** industrial wastes

Water quality parameters are key factors that influence the survival and fitness of living organisms in water bodies (Bald et al. 2005; Pardo et al. 2012; Hussain and Pandit 2012; McDowell et al. 2013). In Burkina Faso, numerous water quality problems have been associated with eutrophication caused by nutrient loading from various sources (e.g., domestic washing, crop production, and cattle waste). Domestic wastes, including inputs of industrial wastes and other uses, are major factors in urban areas that affect negatively the aquatic ecosystem health (Fig. 5e, f). The high concentrations of phosphorus from effluent discharges can cause water quality problems by over-stimulating algal growth that in turn depletes oxygen in the water column. Criteria, such as absence of urban and industrial discharges near to potential reference sites, have to be considered in reference site selection. Other previous studies underlined the importance of physicochemicals for bio-monitoring in tropical streams (Thorne and Williams 1997; Lakew and Moog 2015). Both dissolved oxygen and conductivity, among others measured in water quality assessments, are likely to be affected by different riparian land use types. However, this preliminary approach may help to enrich the debate guiding further study in the region.

### Environmental data sampling

Data was recorded at each sampling site for several variables that are likely to be affected by different riparian land use types and thus reflect human impact on Burkina Faso rivers. Conductivity (μS/cm) and dissolved oxygen (mg/l) were measured with field multimeters (WTW340I). We characterized investigation sites as “reference” or “impaired” based on land use patterns, the degree of habitat degradation as quantified by the protocol (ASSESS-HKH, adapted Susfish 2012), and on variables characterizing hydro-morphological modification (Barbour et al. 1996; Mühlmann 2010; Lakew and Moog 2015) as well as expert judgments. “Experts” include people with a profound knowledge on hydro-biological/limnological topics and a deep insight into local circumstances. Depending on the issue, this may include foresters, rangers, fisheries experts, nature conservation management, ministerial, taxonomic scientists, or hydro-biologists outside the authors’ consortium. For the hydro-morphological characterization, the scoring was conducted using six variables following Mühlmann (2010). Accordingly, a score of 1 was awarded for no or near-to-natural disturbance, 2 for slight disturbance, 3 for moderate disturbance, 4 for strong disturbance, and 5 for heavy disturbance (supplement material Table A). The remaining variable assignments were done by expert consensus following Korte and Moog (2006) visually by means of field protocol (Table 1).

From the variables measured in the field or literatures studies, thirty-seven (37) criteria were selected as plausible by an expert consortium (e.g., ministry; local river authorities, more detailed in Table 2). The criteria were grouped into six categories: status, hydro-morphological features, physicochemical features, sensoric features, land use, and biological elements. These groups were arranged into 37 categories to describe the reference conditions of semi-arid streams and rivers following other authors quoted in the table and considering the particular condition of study area. We proposed 37 a priori criteria that a site has to fulfill to be considered a reference site (Table 2). These 37 criteria include a wide range of human uses and impairments on streams/rivers, and details are given in the previous paragraphs that focus on the four main criteria.

**Table 2:** Summary of the selected criteria for semi-arid streams and rivers

Category	Attributes	Criteria	Conditions	References of tools to be used
Status		1. Protection status	Protected areas	Assemblée Nationale (1997 and 2001)
		2. River bed dynamics	(Near to) natural*)	Mühlmann (2010)
		3. Channel form	(Near to) natural*)	Mühlmann (2010)
		4.Substrate composition	(Near to) natural*)	Mühlmann (2010)
		5. Bank dynamics	(Near to) natural*)	Mühlmann (2010)
Hydro-morphological features		6. In-channel features	(Near to) natural*)	Mühlmann (2010)
	River morphology	7. Channel structure typical to the typology	Near to) natural*)	Hughes (1995)
		8. Dam barrier or reservoir upstream at 500 m of sites	No dam barrier or reservoir**)	Present study
		9. Habitat composition	Representative diversity of substrate composition corresponds to related typology**)	Johnson et al.(2013)
		10.Spawning habitats for the natural fish population	(Near to) natural***)	Barbour et al. (1996)
		12. Sand or gravel excavation	No**)	Nijboer et al. (2004)
	Hydrological condition	13. Alteration of the natural hydrograph and discharge regime	No alteration****)	Barbour et al. (1996)
		14. Water extraction for hydropower and industrial uses	No****)	Present study
		15. Water extraction for irrigation	No (few exception tolerated if in harmony with nature)**)	Hering et al. (2003)
Physicochemical features	Point source pollution	16. Point source pollution and eutrophication	No**), ***)	Hering et al. (2003)
		17. Sign of salinity	No*****)	Present study
		27. Diffuse input	No**)	Nijboer et al. (2004)
Sensoric features		18. Color and odor	Only natural**)*	Moog, and Sharma (2005)
		19. Foam	Only natural***)	Moog, and Sharma (2005)
		20. Turbidity	Only natural***)	Moog and Sharma (2005)
		21. Waste dumping	No**)	Moog and Sharma (2005)
	Physicochemical	22. Conductivity	< 75 μs/cm*****)	Present study
		23. Dissolved oxygen	> 6.0 mg/l*****)	Present study
	Nonpoint source poll.	24. Livestock at 100 m of site	No**)	Present study
		25. Cattle watering	No, only wildlife**)	Lakew et Moog (2015)
	Direct water uses	26. Washing and bathing	Only minimal activities**)	Hering et al. (2003)
Land use		28. Crop farming in the riparian zone	No**)	Hering et al. (2003)
		29. Riparian vegetation	(near to) natural*)	Mühlmann (2010)
		30. Extensive agriculture	No**)	Kaboré et al. (2015)
		31. Intensive agriculture	No**)	Kaboré et al. (2015)
		32. Urbanization, industry, and other uses	No**)	Kaboré et al. (2015)
		33. Fishery activity	No evidence**), ***)	Kaboré et al. (2015)
		34. Human settlement in the floodplain area	No**), ***)	Kaboré et al. (2015)
		35. Riparian zone use for recreation	Occasional**)	Kaboré et al. (2015)
		36. Lateral connectivity between river and riparian zone	Natural**)	Richardson et al. (2012)
Biological elements		37. Presence of wild birds and mammals	Possibly (field observation) **), ***)	Barbour et al. (1996)

*) class 1 of the Mühlmann classification system; **) yes/no-information by field trips or written information, Google earth map; ***) information available from Ministry of Environment and Sustainable Development, local river authority, or other sources (e.g., local fishermen, foresters, natural park guides); ****) information available at the Water and Environment Ministry or written information; *****) in-situ measurements with probes (e.g., conductivity meter; oxygen meter)

## Data analysis

All statistical analyses were performed using the software SPSS version 21 (IBM SPSS 2012) on the key qualitative variables in the study sites to identify major gradients in environmental differences between the sites. In order to select site categories as a basis for a bioassessment program, including reference condition selection, we first conducted hierarchical cluster analysis (Ward linkage methods, Euclidean distance). This analysis was conducted on a qualitative ordinal matrix including all sites. Tested variables were z-standardized prior to the analysis. The significance of cluster support was assessed with a nonparametric test (nonlinear discriminant function analysis) to test the performance of the clusters (% variance). Each cluster is defined by identification category. With the help of the cluster designations, it was possible to show interactions between independent pressures and then to quantify the categories of pressures following the principles described previously by Schinegger et al. (2012) and Stranzl (2014). We defined three main types of pressures indices, hydro-morphological pressures (HydMorPI), water quality pressures (WQPI), and land use pressures (LUPI) following the study setting criteria for the calculation. All calculation was based on averaging the single pressure parameter score adapted (see also, Stranzl 2014; Mostafavi et al. 2015) from the following formulas 1 and 2:

1 $$ \mathrm{Type}\  \mathrm{of}\  \mathrm{pressure}=\frac{\sum_{i=1}^n\mathrm{categories}\  \mathrm{pressure}\  \mathrm{score}}{m} $$

where *m* is the number of pressures, and then we assessed the overall pressure index to have a clear sight in retrospect to human impact, “with affected groups = contributed pressure categories number”, expressed as:

2 $$ \mathrm{Overall}\  \mathrm{pressure}\  \mathrm{index}=\frac{\sum_3^1\mathrm{types}\  \mathrm{of}\  \mathrm{pressure}}{3}\times \mathrm{affected}\  \mathrm{groups} $$

## Results

**Fig. 6:**
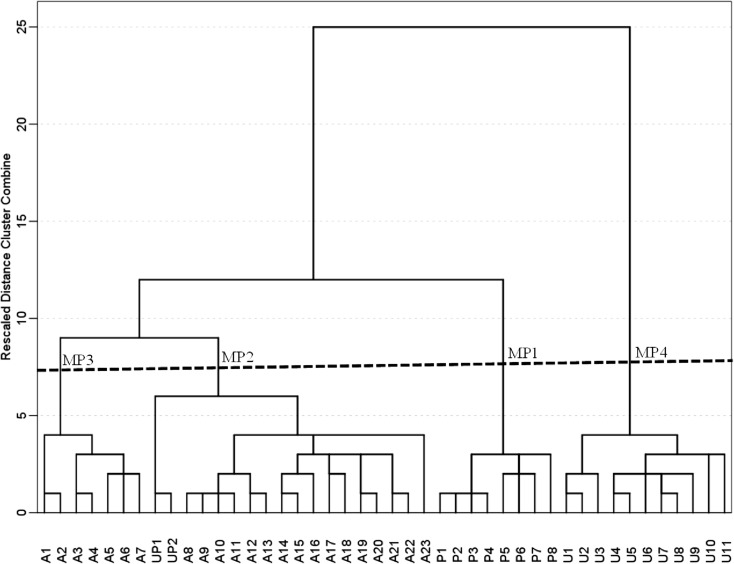
Dendrogram showing the grouping of sites based on human pressures. Four main groups were shown by dendrogram which MP1=protected area (P reference), clustered together MP2 and MP3=extensive agriculture and intensive agriculture (A), and MP4=urban (U). The explained variance of the discriminant analysis test was around 81.8%

The findings of this study are based on a set of variables that were measured in the field. These variables can be grouped according to seven pressures (morphological, habitat, hydrological, longitudinal connectivity, water quality, riparian land use, and direct pressures) that are listed in Table 1. Analysis summarized in Fig. 3 revealed that sites were clustered in four distinct groups, each reflecting a distinct level of pressures. Clusters identified at the lowest, i.e., coarsest, hierarchical level (Fig. 6) corresponded to the four categories formulated based on multiple pressure assessment of study sites: “protected” (MP1 = P1–P8), “intensive agriculture” (MP2 = A1–A7), “extensive agriculture” (MP3 = A8–A23) which included UP1 and 2 in the same category, and “urban” (MP4 = U1–U11). Extensive and intensive agriculture sites (MP2 and MP3, respectively) were then clustered together suggesting their similarity in terms of pressure, while urban areas were found to be the most distinct from all the rest of the sites (MP4). The ordination cluster analysis strongly supported our categorization of sites based on environmental parameters (Fig. A in Electronic supplementary material).

**Fig. 7:**
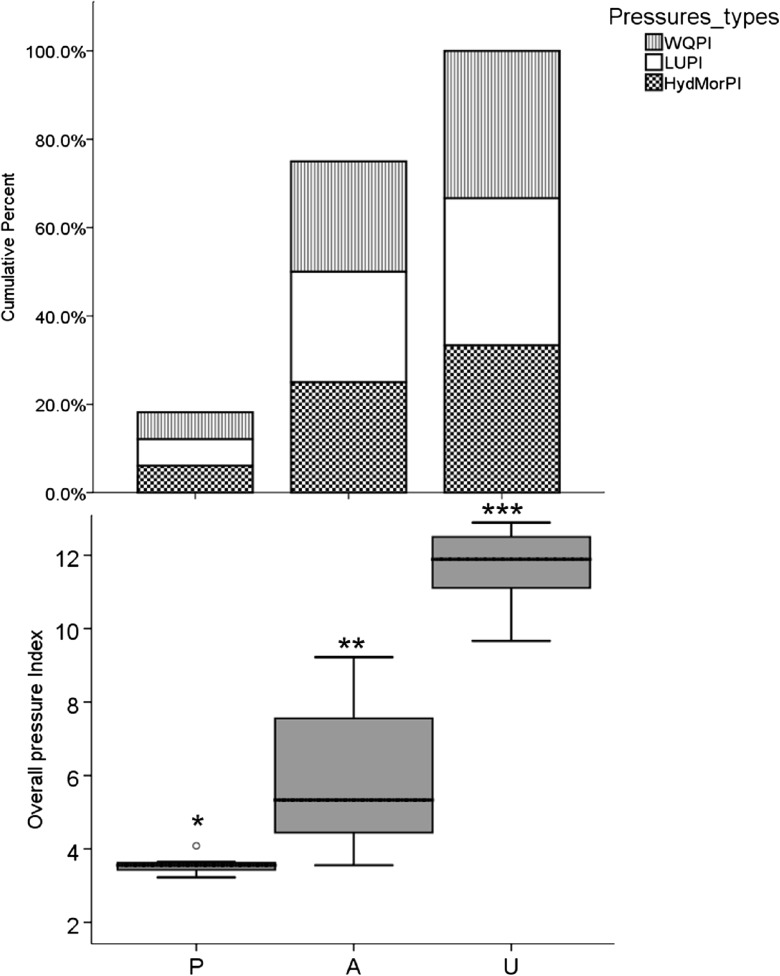
Cumulative percentage of pressure indices per site category and overall pressure index. Stars above box plots indicate statistical significance of differences between site categories (pairwise multiple comparison tests, *p* < 0.05). WQPI: water quality pressure index, LUPI: land use pressure index, HydMorPI: hydromodification pressure index

The cumulative percentage of disturbances assessed in the study sites showed a clear association in environmental parameters with site categories (Fig. 7a). The site categories showed a clear increase across a gradient of human impact intensity in terms of hydro-modification, water quality, and land use (Fig. 7a). Some distinct differences relative to the pressures could be observed in the categories of sites. The lowest intensity of pressure was observed in protected sites (reference “P,” Fig. 7a, b). Hydro-modification, water quality, and land use pressures were represented in “P” areas by a small fraction (less than 20%) constituting a very low overall pressure index (3.56 ± 0.30, Fig. 7a, b), followed by agriculture areas “A.” In contrast, the highest intensity of pressures was found in impacted streams “U” (11.72 ± 0.30, Fig. 7b), e.g., significantly linked with areas affected by human pressures. To simplify further analysis, norms used to assess ranges of pressures were quantified using (1) objective statistical methods, (2) field inspections corresponding to in situ visual evaluations, and (3) expert judgment based on opinions from the scientific community. Thus, in absence of purely pristine sites, protected sites were retained as reference after careful checks of the cumulative effects of pressures.

## Discussion

The use of ecological approaches for managing water resources has so far received little attention in West Africa, especially in Burkina Faso, where water bodies and river systems are strongly impaired by human activities. The presence, diversity, trophic level, density, and biomass of certain fish and benthic invertebrate genera and species respond negatively to a range of anthropogenic pressures (Melcher et al. 2012; Kaboré et al. 2015). We found evidence of three pressure categories to some degree in nearly all study sites. While these pressures can act singly, in most cases, multiple factors act jointly on water quality. Parameters that can reflect the degree of disturbance include water temperature, substrate composition, bank and bed stability, sedimentation rate, physical parameters (e. g., turbidity), and water chemistry (nutrients, contamination). Disturbances of such factors can potentially make the water bodies unsuitable for macrophytes and animals (Aurouet et al. 2005; Munné et al. 2012). As we look from protected areas to urban areas following the coarse categorizations of the study sites, the results show evidence of a gradient of impacts as the number and intensity of multiple anthropogenic pressures accumulate.

While optimal reference sites would represent pristine conditions, this objective is unrealistic in Burkina Faso as it is in most continents in the north of Antarctica. However, in the absence of patently unimpaired sites, a base level of impact must be defined as a reference level. It is therefore important to select representative reference sites that are least disturbed, because the definition of the reference site has important consequences for the development of biological indicators and attainment of threshold values (Hering et al. 2003; Pardo et al. 2012). Here, sites in the protected areas can reasonably be considered as good reference sites as far as they show very low impact levels. These areas show some relatively “natural” characteristics that are hardly distorted by permanent or significant human disturbances. The designation of protected status allows these areas to benefit from better management that preserves near-natural conditions. Both cluster and overall pressure index analyses support strongly our conclusion that protected areas can reliably be used as reference sites and already showed the suitability for ordinating benthic macroinvertebrate communities on a gradient of relative disturbance (Kaboré et al. 2015). The range of different elements used to define such conditions included a wide range of parameters related to the land use, hydro-morphological characteristics, and water quality. Our criteria for selecting the reference sites also generally meet the requirements outlined by other authors (Thorne and Williams 1997; Dallas 2007; Lakew and Moog 2015). Here, as the specified environmental features of a protected area, our findings define criteria for what features should be protected and reinforce the need to maintain a range of protected areas for effective biological reference sites. This study yielded a solid first step toward guidelines that scientists throughout West Africa can now work with to create a single definition of riverine reference conditions. However, Barbour et al. (1999) and Stoddard et al. (2006), among others, argue that even if such a single definition is achieved, these criteria could be varied across ecological regions as the characteristics of the landscape and human use of the landscape. Protected areas enable climate change adaptation and host an important biological diversity crucial for effective conservation of the regional fauna that merits more attention by the competent authorities and scientists. A priori criteria are being increasingly used as cost-effective classification system to calibrate the effects and magnitude of human disturbance on aquatic ecosystems.

## Conclusions for aquatic ecosystem conservation and policy implementation

Running waters are threatened by multiple impacts of human activities, notably by severe pollution and habitat degradation from intense urbanization and agriculture. Legislation in some developing countries, such as Burkina Faso, recognizes that basic human and environmental needs should be met for long-term water ecological services. This study represents the first probe to establish reference condition criteria for the selection of minimally disturbed streams or rivers in the Sahel region and to provide a foundation for ecological status assessment. In view of rising rates of human pressure, the identification of protected areas appears to be crucial for ensuring the long-term sustainability of aquatic biodiversity. This study lays a solid foundation for research to support management that can build and sustain aquatic biodiversity through relatively simple development and application of aquatic biomonitoring tools. Our research demonstrates that such tools could be founded on the reference conditions approach, e.g., a classification system and representative parameters to reflect different degrees of human impact on aquatic ecosystem, for management and conservation of water and river systems in West Africa. The authors would like to encourage African limnologists to use their data on biological quality elements (e.g., algae, macrophytes, benthic macroinvertebrates, and fish) to refine and test the results of this study to help in the further validation of minimally disturbed sites. This procedure may be very helpful for “early warning” information on hotspots for bio-diversity and conservation. Future improvement of these tools requires integrating science and policy to, first, test whether some of the criteria that have been proposed to define reference conditions should be seen as compulsory to classify a site as a reference, and second, for those responsible to formulate and administer policy to commit to long-term monitoring of the integrity of aquatic communities through environmental bio-assessment methods based on the reference condition approach. Learning the effect of disturbances on reference communities can help to guide decision-making about land use and restoration useful for resource managers, conservationists, and politicians to design and enforce appropriate management plans and can help to raise general public awareness for the protection of water bodies.

## Electronic supplementary material

ESM 1 (DOCX 198 kb)
