# Supplementary material for: Developing reference criteria for the ecological status of West African rivers
Source: Environ Monit Assess. 2017 Dec 2;190(1):2. doi: 10.1007/s10661-017-6360-1 (PMC5717125; doi:10.1007/s10661-017-6360-1)
Supplement: Supplementary file 1 — (DOCX 198 kb) [file 10661_2017_6360_MOESM1_ESM.docx]

**ELECTRONIC SUPPLEMENTARY MATERIAL**

Developing reference criteria for the ecological status of West African rivers

Kaboré I.^2^, O. Moog^1^, A. Ouéda^2^, J. Sendzimir ^3^, R. Ouédraogo^4^ , W. Guenda^2^ and A. H. Melcher^1^

^1^ BOKU University of Natural Resources and Life Sciences, Centre for Development Research, Institute of Hydrobiology and Aquatic Ecosystem Management, Vienna, Austria.

^2^ Université de Ouagadougou, Laboratoire de Biologie et Ecologie Animales (LBEA), Burkina Faso.

^3^ International Institute for Applied Systems Analysis (IIASA), Vienna, Austria.

^4^ Ministère de la Recherche Scientifique et de l'Innovation, Institut de l’Environnement et de Recherches Agricoles (INERA), Burkina Faso.

**Fig. A** Ordination groups of investigation sites shown by cluster, which MP1= Protected areas (“P”); MP2-MP3= Extensive and intensive agriculture areas (“A”) and MP4 indicate urban sites (“U”)


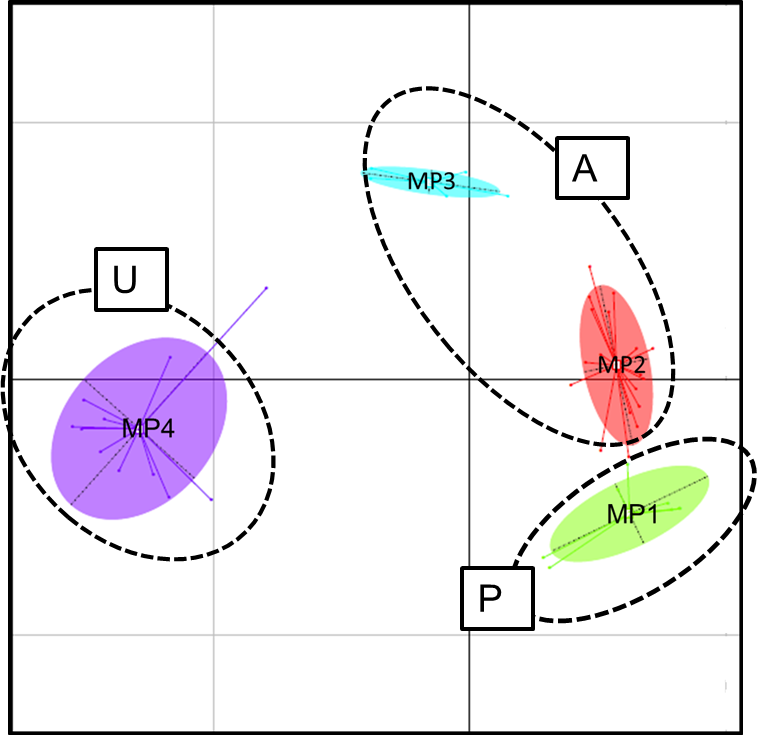


**Table A.** The specific numerical quantitative data of the reference criteria collected in each sampling areas.

P5:Bissiga; P3:Bodjero; P8: Boromo; P4: Guingette; P1: Karfiguela; P2: Kou_foret; P6: Koro1; P7: Koro2; A17: DI; A10: Djaraba; A16: Joint_Nak/Mass; A19: Kougri; A12:Lery; A21: Nagreogo; A23: Niango; A11: Ouéssa; A8: Poweri1; A9: Poweri2; A18: Segda; A14: Toma île; A13: Tengrela1; UP1: Bangreweogo1; UP2: Bangreweogo2; A15: Bissiga2; A22: Kou2; A20: Massili; A5: Boura; A6: Gouran; A3: Korsimoro; A7: Loumbila; A1: Nianssan2; A2: Nianssan2; A4: Peele; U4: Ouaga1; U9: Houet1; U1: Houet2; U10: Tengrela2; U11: Ouaga2; U6: Dioulassoba; U2: Houet3; U7: Kadiogo; U5: Koko; U3: Kua; U8: Nianko. (*) indicates *a priori* additional compulsory information.
